# PancDS in Real‐World Practice: A Prospective Multicenter Validation of a Clinical Decision‐Support System Bridging Experience Gaps in Pancreatic Lesion Diagnosis

**DOI:** 10.1002/advs.75747

**Published:** 2026-05-19

**Authors:** Zhibo Wang, Weinuo Qu, Wenwen Cai, Chuhuai Wang, Chenxi Lyu, Qingguo Xie, Qian Chu, Yaqi Shen, Peng Xiao, Feng Li, Qingpeng Zhang, Jiali Li, Jeong Min Lee, Zhen Li

**Affiliations:** ^1^ Department of Radiology Tongji Hospital, Tongji Medical College Huazhong University of Science and Technology Wuhan China; ^2^ Department of Radiology Shanxi Bethune Hospital Tongji Shanxi Hospital Taiyuan China; ^3^ College of Life Science and Technology Huazhong University of Science and Technology Wuhan China; ^4^ Department of Oncology, Tongji Hospital, Tongji Medical College Huazhong University of Science and Technology Wuhan China; ^5^ Department of Radiology Xiangyang Central Hospital/Affiliated Hospital of Hubei University of Arts and Science Xiangyang China; ^6^ Musketeers Foundation Institute of Data Science The University of Hong Kong Hong Kong China; ^7^ Department of Pharmacology and Pharmacy LKS Faculty of Medicine The University of Hong Kong Hong Kong China; ^8^ Department of Radiology Seoul National University Hospital Seoul South Korea; ^9^ Department of Radiology Seoul National University College of Medicine Seoul South Korea; ^10^ Institute of Radiation Medicine Seoul National University Medical Research Center Seoul South Korea

**Keywords:** artificial intelligence, diagnosis, multicenter study, pancreatic cancer

## Abstract

Distinguishing pancreatic ductal adenocarcinoma (PDAC) from mass‐forming pancreatitis (MFP) is challenging due to imaging mimicry and reader‐dependent variability. PancDS is developed as a biomimetic pancreatic decision‐support system that integrates clinical predictors, a radiomics signature, and self‐developed deep features (PANet). PancDS is enabled by TriFusionNet, an adaptive fusion strategy designed to emulate expert reasoning by dynamically reweighting modalities according to diagnostic relevance. In a retrospective multicenter cohort of 1006 consecutive patients with pathologically confirmed resectable PDAC or MFP (2014–2023), 634 patients from Tongji Hospital were used for training/internal testing, and 372 patients from four independent hospitals for external testing. PancDS achieves internal and external AUCs of 0.936 (95% CI: 0.864–0.993) and 0.881 (95% CI: 0.833–0.924), respectively. In a reader study, PancDS significantly improves diagnostic accuracy and sensitivity, with the largest gains in intermediate and junior radiologists (*P* < 0.001) and minimal case‐level deterioration (1.5–3.9%), functioning as a diagnostic equalizer. In a prospective consecutive cohort (Jan–Oct 2025; n = 151), PancDS achieves an AUC of 0.869 (95% CI: 0.725–0.978) and 94.7% accuracy. This practice‐tested, prospectively evaluated system provides a reliable tool for PDAC–MFP differentiation, potentially informing surgical decision‐making and enhancing diagnostic equity across diverse clinical environments.

## Graphical abstract

A biomimetic artificial intelligence system, PancDS, has been developed to distinguish pancreatic ductal adenocarcinoma from mass‐forming pancreatitis by adaptively integrating clinical data, radiomics, and deep learning features. Validated across multicenter, reader‐study, and prospective settings, PancDS improves diagnostic accuracy, particularly for less‐experienced radiologists, and may serve as a diagnostic equalizer across institutions with different levels of expertise.

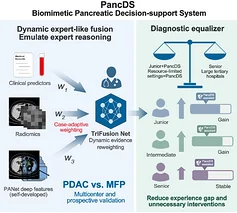

**Abbreviations**
AI: Artificial Intelligence
AUC: Area Under the Curve
CA125: Cancer Antigen 125
CA19‐9: Carbohydrate Antigen 19‐9
CBAM: Convolutional Block Attention Module
CEA: Carcinoembryonic Antigen
CE‐CT: Contrast‐Enhanced CT
CI: Confidence Interval
CNN: Convolutional Neural Network
CT: Computed Tomography
DBIL: Direct Bilirubin
DCA: Decision Curve Analysis
FCN: Fully Connected Neural Network
Grad‐CAM: Gradient‐weighted Class Activation Mapping
HU: Hounsfield Units
IBIL: Indirect Bilirubin
LASSO: Least Absolute Shrinkage and Selection Operator
MFP: Mass‐Forming Pancreatitis
MSD: Medical Segmentation Decathlon
NSFC: National Natural Science Foundation of China
PDAC: Pancreatic Ductal Adenocarcinoma
ROC: Receiver Operating Characteristic
SD: Standard Deviation
TBIL: Total Bilirubin
VITs: Vision Transformers

## 1 Introduction

Pancreatic ductal adenocarcinoma (PDAC) remains a highly lethal malignancy, with a 5‐year survival rate of approximately 9% [1]. While surgical resection offers the only potential for cure, precise preoperative characterization of equivocal pancreatic lesions is clinically challenging [2]. Up to 15%–20% of patients undergoing resection for suspected PDAC are ultimately diagnosed with benign conditions, most commonly mass‐forming pancreatitis (MFP) [3, 4]. This diagnostic ambiguity leads to significant overtreatment, subjecting patients to high‐risk surgeries without oncologic benefit. Therefore, reliable preoperative characterization is critical to avoiding unnecessary surgical morbidity and refining treatment decisions.

This challenge persists because PDAC–MFP differentiation is not a single‐modality pattern‐recognition task, but a context‐dependent clinical reasoning process [5]. In contrast‐enhanced CT (CE‐CT), PDAC and MFP often share overlapping morphologic appearances, making interpretation subjective and strongly dependent on radiologist expertise [3, 6]. Meanwhile, adjunctive evidence is imperfect when considered in isolation: tumor markers such as CA19‐9 lack specificity [7], and biopsy may yield false negatives due to lesion heterogeneity [8]. In practice, expert radiologists mitigate these uncertainties by integrating imaging cues with clinical context and laboratory signals, and by reweighting the importance of each evidence stream depending on the case. However, such experience‐driven integration is unevenly distributed across institutions, contributing to pronounced performance gaps between high‐volume centers and resource‐limited hospitals and ultimately to inequities in care.

Artificial intelligence (AI) could function as a “diagnostic equalizer” by providing objective, quantitative, and standardized assessments [9, 10]. Yet, many existing frameworks remain limited by single‐source inputs; for instance, models relying solely on non‐contrast CT (e.g., the PANDA model) lack the multi‐source evidence integration utilized in practice [11]. Crucially, such reliance on a single modality lacks the necessary redundancy to counteract imaging noise or atypical features, thereby increasing the risk of biased predictions when the primary diagnostic signals are equivocal or inaccurate. Beyond architectural constraints, the scarcity of pathologically confirmed MFP often restricts training to small, single‐center cohorts, undermining real‐world generalizability. This is exemplified by radiomics models that lack external validation, leaving their performance across diverse populations uncertain [12, 13]. Furthermore, a methodological trade‐off persists: while radiomics offers interpretability, it fails to capture the complex, non‐linear semantic features of the tumor microenvironment; conversely, standard ‘black‐box’ deep learning models often lack the context‐aware reasoning required to reconcile discordant clinical and imaging data [14, 15]. Collectively, these constraints highlight a persistent gap between metric‐driven model development and the integrated, context‐aware reasoning deployed by expert radiologists.

To address these limitations, we developed the Pancreatic Decision‐Support system (PancDS) using a biomimetic AI approach. Rather than optimizing isolated algorithmic benchmarks, PancDS replicates the integrated, adaptive reasoning of expert clinicians through an adaptive fusion architecture termed TriFusionNet. By emulating how experts dynamically weigh evidence across discordant imaging and clinical signals, TriFusionNet bridges the inherent gap between interpretability‐focused radiomics and representation‐rich, yet opaque, deep learning. Validated across a large‐scale multicenter framework and a prospective cohort, PancDS functions as a “diagnostic equalizer,” empowering radiologists—particularly those in resource‐limited settings—to achieve expert‐level differentiation of PDAC and MFP. By reducing diagnostic uncertainty and preventing high‐risk, unnecessary surgical interventions, this scalable platform provides a practical solution to promote equitable care and optimize treatment decisions.

## 2 Results

### 2.1 Patient Demographics

The baseline characteristics of patients in the training and internal test cohorts are presented in Table 1. The training cohort (Cohort A) included 376 PDAC patients (mean age, 59.77 years ± 8.19 [SD]) and 67 MFP (mean age, 50.97 years ± 12.35 [SD]) patients. The internal test cohort (Cohort B) comprised 162 PDAC patients (mean age, 59.56 years ± 7.92 [SD]) and 29 MFP patients (51.37 years ± 11.48 [SD]). Compared to the MFP group, patients with PDAC exhibited significantly higher serum CA19‐9 levels in both the training (*P* = 0.001) and test cohorts (*P* = 0.029). Significant elevation of CEA was also observed in the PDAC group within the test cohort (*P* = 0.041). No significant differences were found in other demographic or clinical variables. The clinical and demographic characteristics of the external test cohort (Cohort G) and the prospective cohort (Cohort H) are summarized in Table S1. Cohort H showed no significant differences in age, sex distribution, or laboratory findings, including CA19‐9, amylase, and lipase levels (all *P* > 0.05), indicating well‐matched baseline characteristics. Cohort G exhibited significant differences in age, sex, and CA19‐9 levels (*P* < 0.001), while amylase and lipase levels remained comparable (*P* > 0.05).

**TABLE 1:** Patient demographics.

Characteristics	Training cohort (Cohort A)			Internal test cohort (Cohort B)		
	PDAC	MFP	*P* value	PDAC	MFP	*P* value
Age (years)	59.77 ± 8.19	50.97 ± 12.35	<0.001	59.56 ± 7.92	51.37 ± 11.48	<0.001
Sex			0.051			0.089
Male	214	48		87	23	
Female	162	19		75	6	
CA19‐9 (U/mL)	689.41 ± 1566.62	67.06 ± 249.94	0.001	833.88 ± 1935.71	42.14 ± 61.14	0.029
CEA (ng/mL)	17.01 ± 57.76	5.53 ± 5.31	0.105	14.20 ± 26.65	3.99 ± 3.29	0.041
CA125 (U/mL)	72.82 ± 91.20	63.24 ± 46.30	0.401	58.83 ± 44.31	52.99 ± 30.44	0.497
Amylase (U/L)	95.51 ± 136.30	64.27 ± 66.51	0.067	94.24 ± 163.51	77.34 ± 81.51	0.587
Lipase (IU/L)	288.43 ± 518.33	163.46 ± 215.02	0.052	258.24 ± 545.96	177.04 ± 248.12	0.443
TBIL (µmol/L)	36.26 ± 58.54	23.97 ± 34.03	0.096	36.87 ± 51.07	47.31 ± 165.57	0.514
DBIL (µmol/L)	27.53 ± 53.85	15.96 ± 29.28	0.085	28.78 ± 45.51	29.23 ± 106.60	0.97
IBIL (µmol/L)	8.22 ± 8.93	7.03 ± 5.37	0.29	7.67 ± 6.82	5.80 ± 2.41	0.147

*Note*: CA19‐9 = Carbohydrate Antigen 19‐9, CEA = Carcinoembryonic Antigen, CA125 = Cancer Antigen 125, TBIL = Total Bilirubin, DBIL = Direct Bilirubin, IBIL = Indirect Bilirubin, PDAC = Pancreatic Ductal Adenocarcinoma, MFP = Mass‐Forming Pancreatitis.

### 2.2 Importance Analysis of Clinical Predictors

Integrated gradient analysis identified CA19‐9 as the most influential clinical variable for differentiating pancreatic ductal adenocarcinoma from mass‐forming pancreatitis, followed by age, lipase, sex, and amylase. These five highest‐ranking predictors were incorporated into the clinical component of the final PancDS model. The complete importance ranking is presented in Figure S1 in Section S1.

### 2.3 Comparative Performance of PANet and Baseline 3D‐ResNet50

**FIGURE 1:**
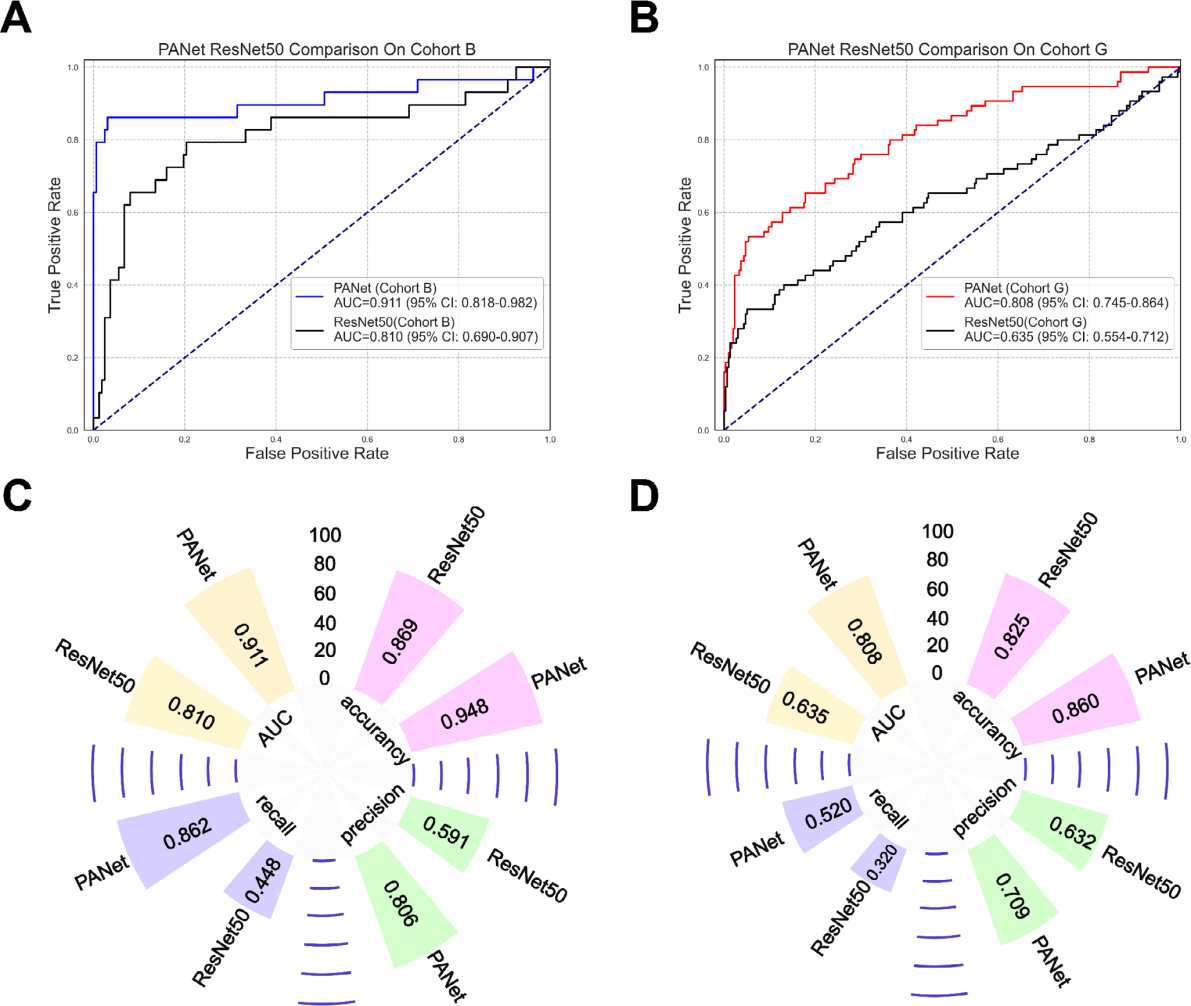
Comparison of PANet and Baseline 3D‐ResNet50 Performance. (A, C) performance of PANet vs. 3D‐ResNet50 in the internal test cohort (Cohort B); (B, D) performance of PANet vs. 3D‐ResNet50 in the external test cohort (Cohort G). Class labels were defined as PDAC = 0 and MFP = 1. AUC = Area Under the Curve, CI = Confidence Interval.

In this study, we encoded the outcome as PDAC = 0 and MFP = 1; therefore, the predicted probability corresponds to MFP (label = 1). Compared with the baseline 3D‐ResNet50 model, PANet demonstrated superior diagnostic performance on both the internal and combined external test cohorts (Cohort G). In the internal test cohort, PANet achieved an AUC of 0.911 (95% CI: 0.818–0.982), significantly outperforming the ResNet50 model's AUC of 0.810 (95% CI: 0.696–0.909; *P* < 0.001). The performance gap was more pronounced in Cohort G, where PANet maintained a robust AUC of 0.808 (95% CI: 0.745–0.864), whereas the ResNet50 model's performance decreased to an AUC of 0.635 (95% CI: 0.554–0.712). These results confirm the superior accuracy and generalizability of the PANet architecture (Figure 1).

### 2.4 Unimodal Model Performance

**FIGURE 2:**
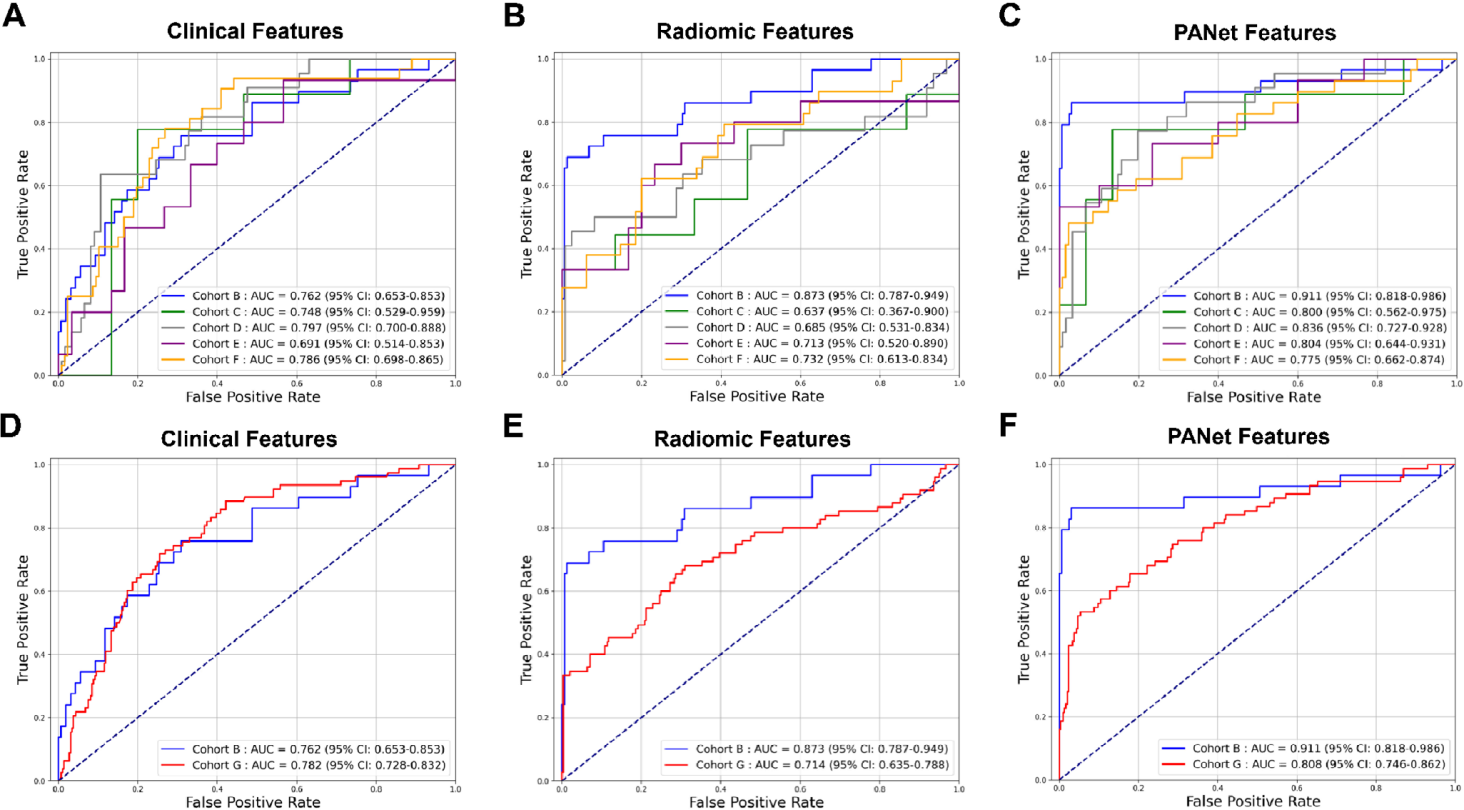
Performance of the unimodal models. (A–C) Performance of each model across the four individual external test cohorts (Cohorts C, D, E, and F) and the internal test cohort (Cohort B). (D‐F) Comparative performance of each model between the internal test cohort (Cohort B) and the combined external test cohort (Cohort G). Class labels were defined as PDAC = 0 and MFP = 1. ROC = receiver operating characteristic, AUC = Area Under the Curve, CI = Confidence Interval.

The performance of each model in each external center is shown in Figure 2. Among the three modalities, the deep learning‐based PANet model demonstrated the highest AUCs, which achieved an AUC of 0.911 (95% CI: 0.818–0.982) in the internal test cohort (Cohort B) and maintained a strong AUC of 0.808 (95% CI: 0.746–0.864) in Cohort G. In contrast, the radiomics model performed well on Cohort B (AUC = 0.873, 95% CI: 0.787–0.949) but showed a substantial performance decrease on the external cohort (AUC = 0.714, 95% CI: 0.635–0.788). The model based on clinical features alone yielded the most modest results, with stable but limited performance across both Cohort B (AUC = 0.762, 95% CI: 0.653–0.853) and Cohort G (AUC = 0.782, 95% CI: 0.728–0.832).

### 2.5 Performance of PancDS Based on TriFusionNet

Performance comparisons of alternative fusion strategies are provided in Table S2. The PancDS tri‐modality framework demonstrated robust discrimination in Cohort B (AUC = 0.936, 95% CI: 0.864–0.993), notably outperforming single‐modality approaches. Cohort G maintained robust performance (AUC = 0.881, 95% CI: 0.833–0.924) and PancDS achieved accuracies of 0.93 and 0.87 in the internal and external validation cohorts, respectively, reflecting comparable classification performance across datasets. The diagnostic performance of the unimodal models and the final PancDS is summarized in Table 2. Moreover, we conducted subgroup analyses stratified by sex, age, and tumor size, which showed consistent performance of PancDS across subgroups (Section S2, Figure S2).

**TABLE 2:** Performance of different models.

Cohorts	Metrics	Clinical model	Radiomics model	PANet model	PancDS
Internal test cohort (cohort B)	AUC (95% CI)	0.762 (0.653–0.853)	0.873 (0.787–0.949)	0.911 (0.818–0.982)	0.936 (0.864–0.993)
	Accuracy	0.81	0.93	0.92	0.93
	Sensitivity	0.48	0.69	0.86	0.86
	Precision	0.39	0.83	0.69	0.71
	Specificity	0.86	0.98	0.93	0.94
External test cohort (cohort G)	AUC (95% CI)	0.782 (0.728–0.832)	0.714 (0.635–0.788)	0.808 (0.745–0.864)	0.881 (0.833–0.924)
	Accuracy	0.78	0.86	0.86	0.87
	Sensitivity	0.35	0.33	0.52	0.68
	Precision	0.47	0.93	0.71	0.69
	Specificity	0.90	0.99	0.95	0.92

*Note*: Class labels were defined as PDAC = 0 and MFP = 1. AUC = Area Under the Curve, CI = Confidence Interval.

**FIGURE 3:**
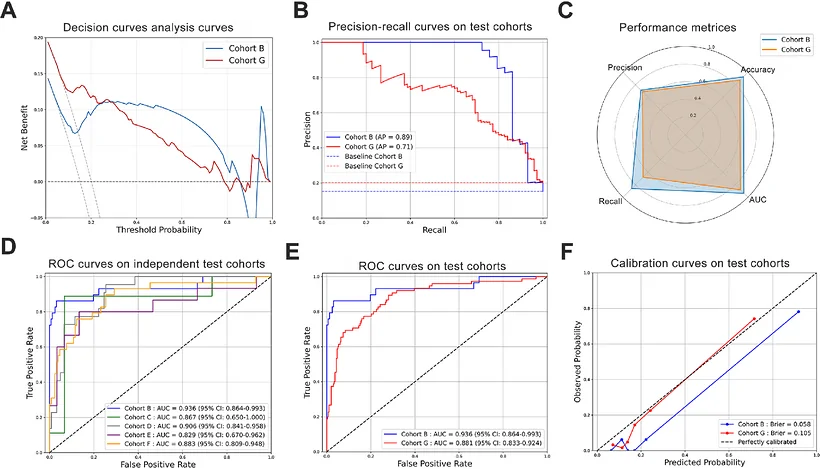
Performance of the multimodal PancDS system based on TriFusionNet. (A) Decision curve analysis; (B) Precision–recall curves; (C) Radar chart of key performance metrics; (D) ROC curves on the internal test cohort (Cohort B) and 4 independent external cohorts; (E) ROC curves on Cohort B and the combined external test cohort (Cohort G). (F) Calibration curves on Cohort B and Cohort G. Class labels were defined as PDAC = 0 and MFP = 1. ROC = Receiver Operating Characteristic, AUC = Area Under the Curve, CI = Confidence Interval.

DCA demonstrated the PancDS's clinical superiority across the critical threshold probability spectrum (0.1–0.85). Particularly within the clinically actionable 0.2–0.8 probability threshold, the multimodal integration demonstrated enhanced decision‐making utility with 0.02–0.12 net benefit increments compared to isolated modality approaches (Figure 3). Calibration curves for Cohort B and Cohort G were closely aligned with the diagonal, with Brier scores of 0.058 and 0.105, respectively.

### 2.6 Model Interpretability Analysis

**FIGURE 4:**
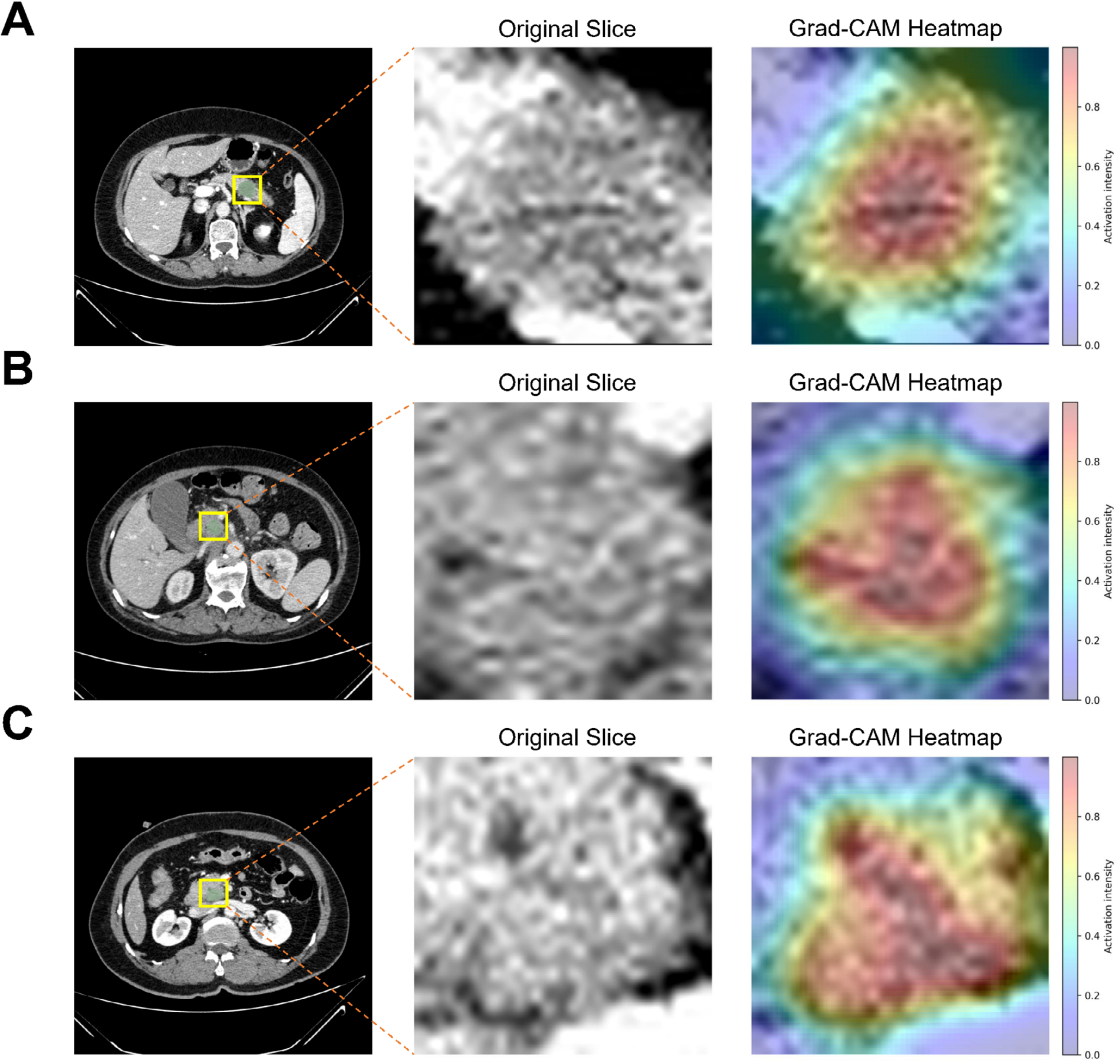
Visualization of model interpretability using Grad‐CAM heatmaps. (A) A 61‐year‐old female patient with MFP. (B) A 64‐year‐old female patient with PDAC. (C) A 64‐year‐old male patient with PDAC. For each case, the original CT slice (left), a magnified view of the lesion (middle), and the Grad‐CAM heatmap overlaid on the magnified view (right) are shown. MFP = Mass‐forming Pancreatitis, PDAC = Pancreatic Ductal Adenocarcinoma, Grad‐CAM = Gradient‐weighted Class Activation Mapping.

Grad‐CAM heatmapping analysis revealed differential attention patterns in lesion discrimination (Figure 4). The model exhibited concentrated activation precisely within pathological regions of both PDAC and MFP cases, while demonstrating minimal response in adjacent pancreatic parenchyma. Thus, the model's activation patterns mirrored radiologists’ diagnostic focus.

### 2.7 Reader‐Study Evaluation of PancDS Utility

**FIGURE 5:**
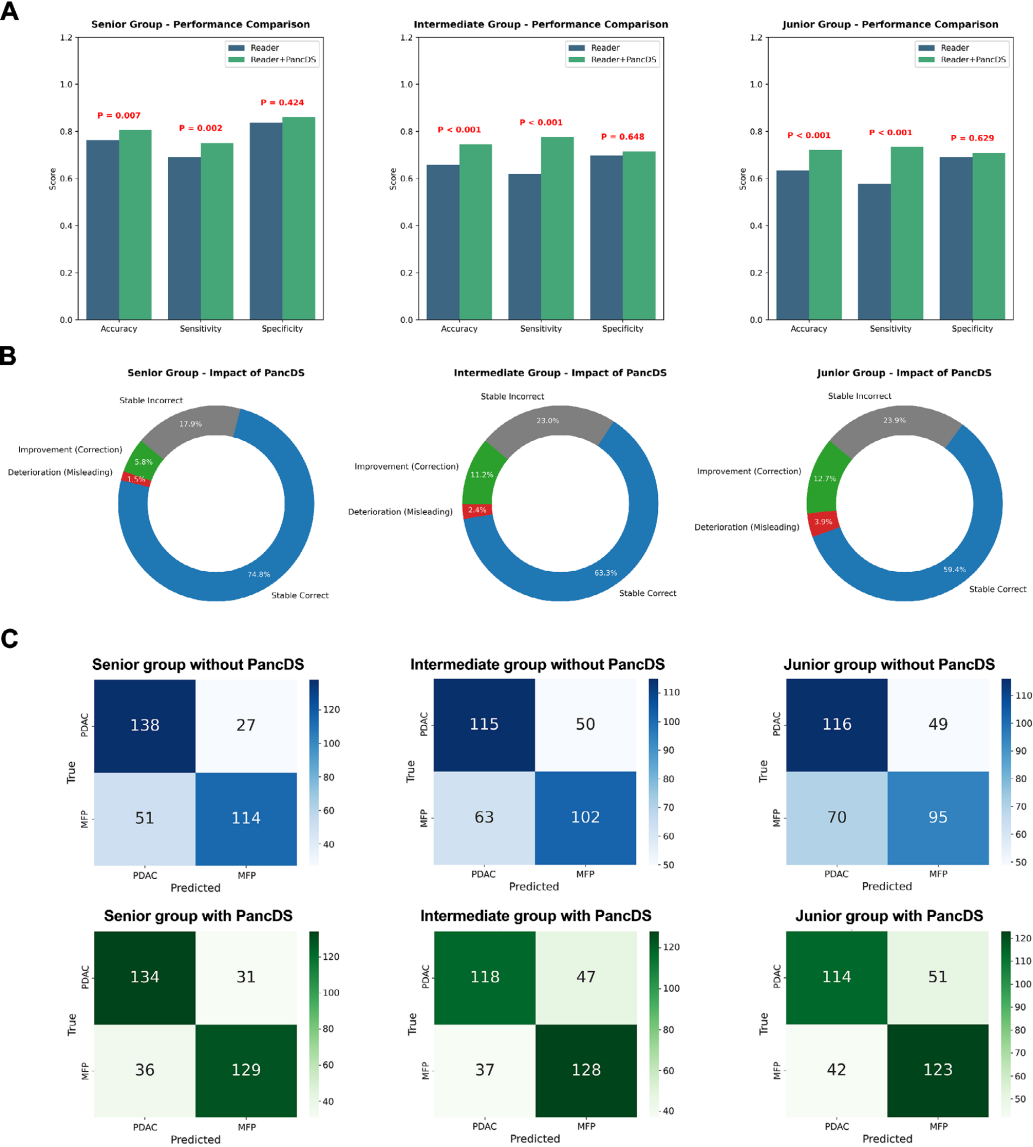
Reader study results with and without PancDS assistance. (A) Accuracy, sensitivity, and specificity for senior/intermediate/junior radiologists unaided vs PancDS‐assisted; P values from McNemar's test. (B) Case‐level transitions: stable correct, stable incorrect, improvement (incorrect→correct), and deterioration (correct→incorrect). (C) Confusion matrices for unaided versus PancDS‐assisted readings. Class labels were defined as PDAC = 0 and MFP = 1.

In the reader study stratified by experience, PancDS assistance significantly improved accuracy and sensitivity across experience strata, with the largest improvements in intermediate and junior radiologists (both *P* < 0.001 for accuracy and sensitivity), while specificity did not change significantly in any group (all *P* > 0.4) (Figure 5A, Table 3). Case‐level analysis further supported these findings (Figure 5B). With PancDS‘s assistance, the proportion of corrected cases (incorrect→correct) was highest in intermediate (11.2%) and junior (12.7%) readers, compared with 5.8% in senior readers. PancDS‐induced deterioration (correct→incorrect) was infrequent across groups (1.5–3.9%), indicating that the net effect of PancDS was predominantly corrective rather than misleading. Confusion matrices for unaided versus PancDS‐assisted readings are shown in Figure 5C, illustrating the corresponding shifts in misclassification patterns across experience groups.

**TABLE 3:** Comparison of diagnostic performance of radiologists with different clinical experience with and without the assistance of the PancDS system.

Group	Metrics	Unassisted (%)	PancDS‐assisted (%)	*P* value
Senior Group	Accuracy	76.36	80.61	0.007
	Sensitivity	69.09	75.15	0.002
	Specificity	83.64	86.06	0.424
Intermediate Group	Accuracy	65.76	74.55	< 0.001
	Sensitivity	61.82	77.58	< 0.001
	Specificity	69.70	71.52	0.648
Junior Group	Accuracy	63.33	72.12	< 0.001
	Sensitivity	57.58	73.33	< 0.001
	Specificity	69.09	70.91	0.629

*Note*: Class labels were defined as PDAC = 0 and MFP = 1.

### 2.8 Prospective Validation

**FIGURE 6:**
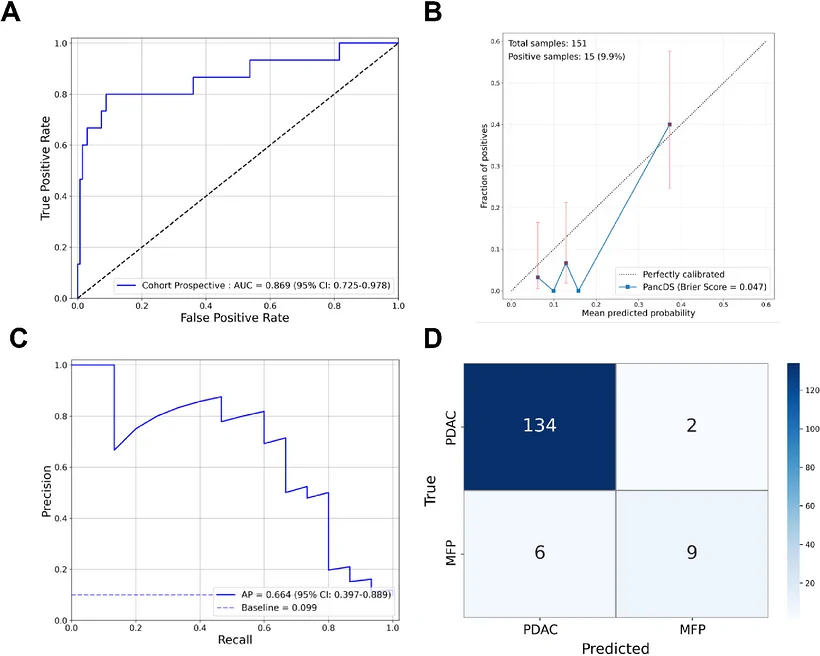
Prospective evaluation of PancDS. (A) ROC curve on the prospective test cohort (Cohort H); (B) Calibration curve on Cohort H. (C) Precision–recall curve on Cohort H. (D) Confusion matrix plot. Class labels were defined as PDAC = 0 and MFP = 1. ROC = Receiver Operating Characteristic, AUC = Area Under the Curve, CI = Confidence Interval.

In the prospective cohort (Cohort H; n = 151, including 136 PDAC and 15 MFP), PancDS demonstrated good discrimination for differentiating PDAC from MFP, achieving an AUC of 0.869 (95% CI: 0.725–0.978). Using the pre‐specified operating threshold, the model yielded an overall accuracy of 94.7%, with a sensitivity of 60.0%, specificity of 98.5% and precision of 81.8%. The corresponding confusion matrix and calibration plot are shown in Figure 6.

## 3 Discussion

In this study, we developed PancDS, a clinical decision‐support framework that integrates deep learning signature, radiomics, and clinical parameters to emulate expert‐level reasoning for pancreatic lesion characterization. Our findings demonstrate that biomimetic PancDS functions as a robust ‘second opinion,’ enhancing radiologist performance with superior accuracy over unimodal models (AUC: 0.936 internal; 0.881 external). Crucially, the system also benefited less‐experienced radiologists, suggesting potential value in narrowing experience‐related performance differences across institutions. Importantly, PancDS also maintained encouraging performance in a prospective consecutive cohort, achieving an AUC of 0.869 (95% CI: 0.725—0.978). To our knowledge, this represents the largest study validating a workflow‐compatible, CT‐based decision‐support tool with prospective evaluation, highlighting its potential to reduce misdiagnosis and guide precise management.

The cornerstone of our feature extraction is PANet, a 3D ResNet‐based architecture that uses a triple‐attention mechanism to intelligently recalibrate feature relevance. This integrated strategy includes channel attention to highlight informative textural patterns of lesions, spatial attention to focus on diagnostically hypoattenuating regions, and a lesion‐aware mask attention that leverages segmentation priors to concentrate computational resources. The introduction of the lesion‐aware mask provides explicit, top‐down guidance, marking a key advance over purely bottom‐up modules like the Convolutional Block Attention Module (CBAM) [16]. This sophisticated, CNN‐native architecture offers an efficient alternative to computationally intensive hybrid approaches like Vision Transformers (ViTs) [17]. These innovations could account for PANet's superior performance over the ResNet.

The efficacy of PancDS is underpinned by the adaptive fusion strategy of its TriFusionNet architecture, which is designed to emulate how expert radiologists synthesize multimodal evidence. Traditional static fusion methods often suffer from a rigid, ‘one‐size‐fits‐all’ limitation, which can dilute critical signals by assigning undue weight to less informative or noisy inputs [18]. In contrast, our model employs a dynamic, contribution‐weighted mechanism to prioritize the most reliable data stream in a context‐aware manner. Specifically, it amplifies clinical signals when imaging is inconclusive while relying more heavily on radiological features when clinical markers are equivocal.

Importantly, PancDS addresses a different clinical question than screening‐oriented unimodal detection systems such as PANDA, which is optimized for opportunistic PDAC detection on non‐contrast CT with high sensitivity [11]. PancDS is instead engineered for the more granular task of preoperative differentiation between PDAC and MFP. Accordingly, with MFP defined as the positive class, our results reveal a deliberate trade‐off: to prioritize oncologic safety, the system operates at a high‐specificity threshold (98.5%) that minimizes the misclassification of PDAC as benign inflammation. While this results in a conservative sensitivity (60.0%) for MFP identification, the corresponding high precision (81.8%) ensures that any ‘benign’ prediction is highly reliable. This performance profile, validated across five institutions over a nearly decade‐long period with robust generalizability (external AUC 0.881), addresses the “implementation gap” of rigorous prospective validation identified in recent literature [19]. By prioritizing a confirmatory decision‐support role, PancDS may provide a reliable signal for conservative management, potentially offering health‐economic benefits by helping to reduce avoidable high‐risk resections and their associated morbidity.

To ensure transparency in the system's decision‐making, we employed Grad‐CAM analysis. The resulting activation maps exhibited high concordance with the pathological core of lesions, mirroring a radiologist's diagnostic focus on lesion boundaries and peri‐pancreatic‐duct changes [20]. Moreover, these activation patterns corresponded to established radiological signs, such as the “duct cutoff sign” in PDAC and the “sausage‐shaped” morphology in MFP [21, 22]. This anatomical consistency between algorithmic attention and radiological semantics provides the requisite credibility to foster physician trust and facilitate clinical adoption.

Crucially, the clinical utility of PancDS was rigorously validated through a real‐world radiologist reader study. We found that less‐experienced radiologists showed substantial improvement in diagnostic accuracy and sensitivity (both *P* < 0.001). The balanced case composition used in the reader study improved statistical efficiency for comparing unassisted and AI‐assisted reading, particularly for MFP, but may limit direct extrapolation of absolute reader‐performance estimates to routine clinical prevalence settings. This finding aligns with prior evidence that AI assistance can disproportionately enhance performance among junior clinicians [23, 24, 25]. These gains are particularly significant given the global shortfall of diagnostic specialists and the widening gap between imaging demand and workforce capacity [26]. Practically, PancDS offers standardized assistance that stabilizes decision‐making where subspecialty expertise is limited. By facilitating the transfer of diagnostic insights from tertiary centers to peripheral settings, this tool could help bridge performance gaps between junior and senior radiologists and promote more equitable access to high‐quality diagnosis. These findings support the potential clinical value of PancDS as a diagnostic decision‐support tool, particularly in reducing experience‐related variability in radiologic interpretation.

Our study has several limitations that should be acknowledged and addressed in future work. First, its reliance on a predominantly Asian population may restrict the broad generalizability, requiring prospective validation across diverse ethnic groups. In addition, although PancDS demonstrated consistent performance across five institutions over a nine‐year temporal span, further validation using more diverse imaging equipment and acquisition parameters is needed to fully establish its technical generalizability. Second, the prospective validation was observational and limited to pathologically confirmed cases to ensure a reliable reference standard. This may introduce selection bias and limit the representation of routine clinical practice. Moreover, the prospective cohort was relatively small, particularly for MFP, thereby limiting the statistical robustness of the prospective estimates. Thus, although PancDS showed promising performance, its clinical value, especially with regard to diagnostic decision‐making, treatment planning, and patient outcomes, has not yet been fully established and warrants further evaluation in larger‐scale prospective interventional studies. Third, other tumors and advanced PDAC with obvious vascular invasion were excluded to focus on resectable cases where the PDAC–MFP dilemma is most clinically impactful. This design may cause spectrum bias and limit generalizability; accordingly, PancDS should be interpreted as an assistive tool for differentiating resectable PDAC and MFP rather than a universal model for all pancreatic tumors. Fourth, our analysis used single‐phase CT data, which do not capture key dynamic contrast enhancement patterns. Future work should therefore focus on expanding disease coverage, integrating multi‐phase CT data, and pursuing further validation and optimization across more institutions.

In conclusion, we developed, multicenter‐validated, and preliminarily prospectively evaluated PancDS, a robust and interpretable decision‐support system for distinguishing PDAC from MFP. Using a biomimetic design, PancDS emulates expert radiologists’ integrated, context‐aware reasoning by adaptively fusing clinical variables, radiomics, and deep learning signatures in a case‐specific manner. Importantly, PancDS has the potential to serve as a diagnostic equalizer—providing a consistent “second‐opinion” standard that narrows expertise‐related performance gaps and supports reliable diagnosis in resource‐limited settings. As a scalable and workflow‐compatible tool, PancDS has the potential to reduce diagnostic uncertainty, avoid unnecessary interventions, and advance more equitable, precision‐guided care for patients with pancreatic masses.

## 4 Experimental Section

### 4.1 Patient Enrollment

**FIGURE 7:**
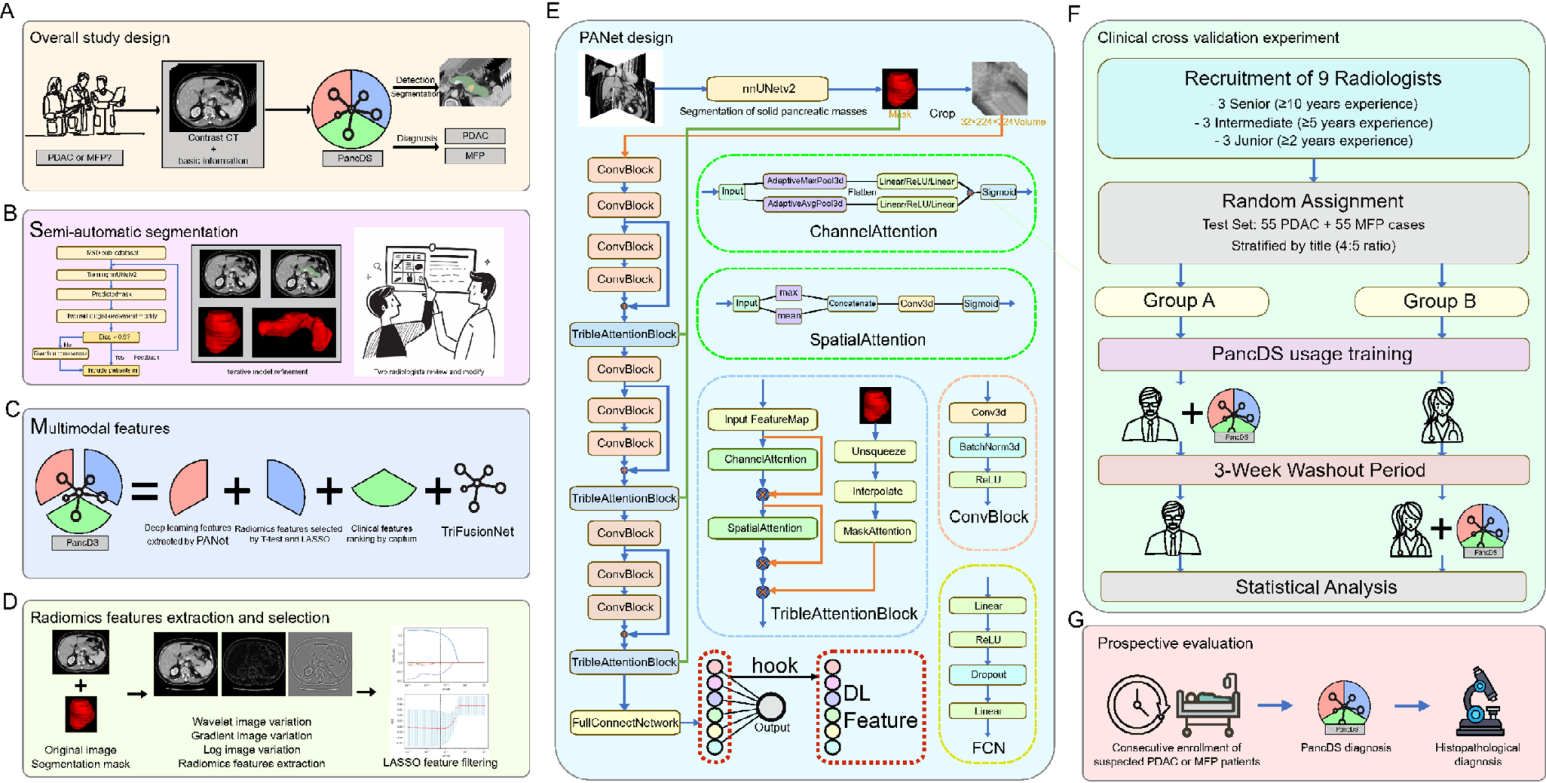
Overview of the development, validation, and clinical evaluation of the PancDS system. (A) Overall study design. The workflow illustrates patient recruitment, data processing using the PancDS system, and the generation of diagnostic predictions for PDAC and MFP. (B) Semi‐automatic segmentation. An iterative model refinement strategy was employed for segmenting the pancreas and solid lesions, with all segmentations reviewed and refined by two experienced radiologists. (C) Multimodal features. PancDS system integrates deep learning features from PANet, radiomics features, and clinical predictors using the TriFusionNet architecture. (D) Radiomics features extraction and selection. The radiomics pipeline involved feature extraction from venous‐phase CT images, followed by a two‐stage feature selection process using an independent t‐test and LASSO regression. (E) PANet design. Schematic of the PANet architecture, a 3D deep learning network designed for pancreatic lesion feature extraction, which incorporates triple‐attention blocks. (F) Clinical cross‐validation experiment. Workflow of the cross‐over reader study designed to evaluate the impact of PancDS on the diagnostic performance of radiologists with varying levels of experience. (G) Prospective evaluation. MFP = Mass‐Forming Pancreatitis, PDAC = Pancreatic Ductal Adenocarcinoma.

This multicenter study was approved by the Institutional Review Board of our hospital (Approval No. TJ‐IRB202506060). Informed consent was obtained for the prospective cohort, and waived for the retrospective cohort. All patient data were de‐identified. The study overview is displayed in Figure 7.

**FIGURE 8:**
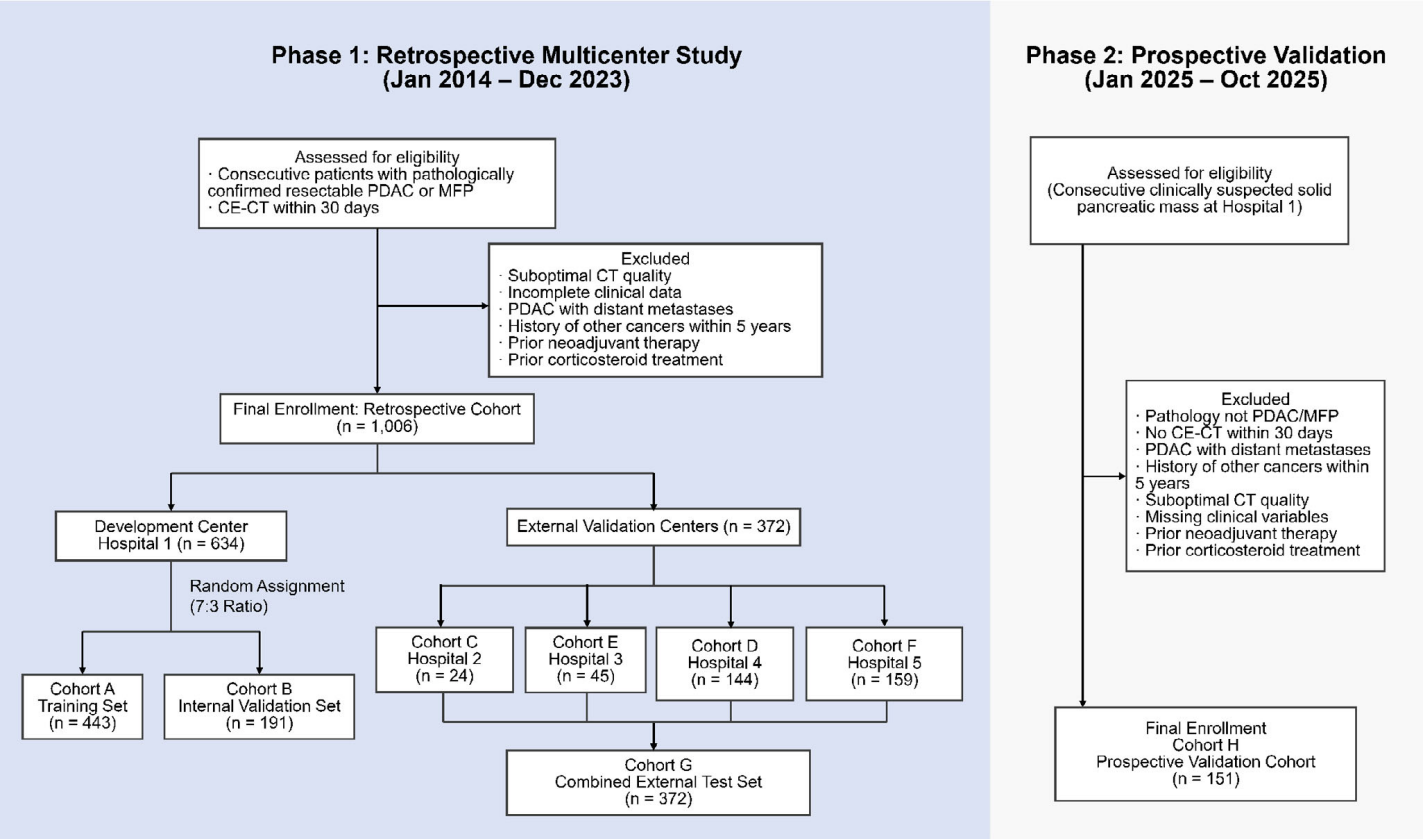
Patient enrollment. To ensure robust model development and assessment, the cohort from (Hospital 1; n = 634) was divided into training (Cohort A; n = 443) and internal test (Cohort B; n = 191) sets using stratified random sampling (7:3 ratio, stratified by PDAC/MFP outcome). The independent cohorts from the four additional centers (Cohorts C‐F) were pooled to create a held‐out external test cohort (Cohort G; n = 372). Hospital 1 = Tongji Hospital, Tongji Medical College, Huazhong University of Science and Technology, Hospital 2 = Liyuan Hospital, Tongji Medical College, Huazhong University of Science and Technology, Hospital 3 = Peking Union Medical College Hospital, Hospital 4 = Shanxi Bethune Hospital, Hospital 5 = Xiangyang Central Hospital, PDAC = Pancreatic Ductal Adenocarcinoma, MFP = Mass‐Forming Pancreatitis.

Between January 2014 and December 2023, we enrolled 1006 consecutive patients with pathologically confirmed resectable PDAC or MFP from five medical centers. Eligible patients were required to have undergone preoperative contrast‐enhanced CT within 30 days before surgery and have complete clinical records available. Detailed criteria and information are shown in Figure 8 and Section S3.

### 4.2 Clinical Predictor‐Based Model

We initially evaluated 10 clinical features relevant for differentiation: age, sex, carbohydrate antigen 19‐9 (CA19‐9), carcinoembryonic antigen (CEA), carbohydrate antigen 125 (CA125), pancreatic amylase, lipase, total bilirubin, direct bilirubin, and indirect bilirubin [27]. We applied Captum's Integrated Gradients method to quantify feature importance, moving beyond univariate significance to identify key diagnostic predictors (Section S1) [28]. The top five identified features were subsequently used to develop the clinical model based on a fully connected neural network architecture (FCN) (Figure S1).

### 4.3 CT Image Acquisition

All patients underwent contrast‐enhanced CT on multidetector scanners at the participating centers. Different scanner models were used across institutions. Detailed information on the major scanner models used at participating centers is provided in Table S3. A common pancreatic CT protocol framework was followed, including a 2.5–3.0 mL/s injection of iodinated contrast (iopromide, 370 mgI/mL; 1.0–1.5 mL/kg), bolus‐tracking with a threshold of 120 HU, pancreatic phase acquisition at approximately 40–50s after contrast injection, and portal venous‐phase acquisition at approximately 60–70s. Acquisition parameters were: 120 kV tube voltage, 1 mm slice thickness, 1.25 mm reconstruction interval, 512×512 matrix, 0.984:1 pitch, 0.5‐second gantry rotation time.

### 4.4 Semi‐Automatic Segmentation

Lesion segmentation was performed using an iterative, semi‐automated workflow. An initial segmentation mask was generated by a nnUNetv2 model pre‐trained on the MSD dataset and then independently reviewed and manually refined by two experienced radiologists, who were blinded to the final pathological diagnoses. Masks with Dice > 0.90 were retained, while the remainder underwent a consensus review (Section S4 and Figure S3).

### 4.5 Radiomics Signature Construction

Radiomics features were extracted from venous‐phase CT images using Pyradiomics (v3.0.1). Venous phase was selected as it provides optimal pancreatic parenchymal enhancement for lesion characterization and is universally acquired in pancreatic protocols. Before extraction, images were normalized and resampled to 1×1×1 mm^3^ spacing via B‐Spline interpolation, with a fixed bin width of 25 HU. Full configuration details are available in Section S5. This process yielded 1236 features, encompassing morphological descriptors, first‐order statistics, and advanced texture metrics. After cohort‐specific standardization, a two‐stage selection process was employed: univariate t‐test (*P* < 0.05) reduced 1236 features to 15 candidates, followed by LASSO regression with 10‐fold cross‐validation to identify the six most robust features for the final signature. The six selected radiomic features included original_shape_Maximum2DdiameterSlice, original_glcm_JointAverage, log‐sigma‐3‐0‐mm‐3D_glrlm_RunLengthNonUniformity, log‐sigma‐3‐0‐mm3D_glszm_GrayLevelNonUniformity, wavelet‐LLL_glszm_GrayLevelNonUniformity; and gradient_glcm_Imc2. The radiomic signature was built with these selected features with the FCN.

### 4.6 PANet Signature Construction

We developed PANet, a 3D deep learning feature extractor designed to model the multilevel imaging characteristics of pancreatic lesions. The network incorporates a triple‐attention subsystem to enhance feature discriminability. Specifically, Channel and Spatial attention modules were integrated to adaptively recalibrate feature weights across dimensions and highlight diagnostically relevant regions. Crucially, a Lesion‐Aware attention module was employed to embed segmentation priors, explicitly directing the model's focus toward tumor boundaries and internal heterogeneity. The PANet aggregates multiscale features through adaptive pooling and extracts 64‐dimensional deep signatures. This compact representation mirrors the progressive visual reasoning used by expert radiologists and serves as the foundational imaging input for the subsequent multi‐modal fusion stage (Section S6, Figure S4). To evaluate the efficacy of PANet, we benchmarked its performance against 3D‐ResNet50, a widely recognized and well‐established baseline architecture in the field of 3D image classification.

### 4.7 Multimodal Feature Fusion and PancDS

We developed PancDS, a multimodal diagnostic model for differentiating PDAC from MFP. The core architecture of PancDS is TriFusionNet, which integrates three complementary inputs: selected clinical predictors, radiomics features, and deep‐learning features extracted by PANet. TriFusionNet employs a contribution‐aware weighting scheme that dynamically adjusts the influence of each modality according to its diagnostic relevance. Specifically, the model can up‐weight clinical information when imaging evidence is inconclusive and place greater emphasis on imaging‐derived features when clinical markers are uncertain. The modality‐specific feature spaces are subsequently projected into a unified 256‐dimensional representation that preserves complementary signals while enhancing the combined discriminative strength (Section S7 and Figure S5). We evaluated candidate cut‐offs across the ROC curve and selected the threshold that maximized Youden's J index while prioritizing high specificity to minimize misclassification of PDAC as benign disease. This reflects the clinical imperative of oncologic safety: ensuring that patients with true malignancy are not inappropriately diverted from curative resection. The resulting threshold (0.4) was fixed for all subsequent test cohorts and prospective evaluation. The code is available on https://github.com/BratherKISKIS/PancDS.

### 4.8 Model Interpretability Analysis

We implemented the gradient‐weighted class activation mapping (Grad‐CAM) method to demystify decision‐making processes within the PancDS. By applying this visualization technique to convolutional layers, we generated class activation heatmaps that reveal the model's focal regions on CT scans.

### 4.9 Cross‐Validation of PancDS in Assisting Radiologists with Clinical Diagnosis

To evaluate the clinical utility of the PancDS in real‐world practice, we conducted a cross‐over reader study with nine radiologists, stratified by experience into three groups: senior (n = 3, ≥10 years’ experience), intermediate (n = 3, 5–10 years’ experience), and junior (n = 3, 2–5 years’ experience). Here, “real‐world practice” refers to evaluation using routine clinical data from actual patients across multiple institutions, rather than relying solely on retrospective algorithmic performance assessment. This reader study was performed in a controlled observer‐study setting designed to approximate routine radiologic interpretation, rather than as a real‐time deployment study embedded in clinical workflow. 110 cases were randomly selected from the external test cohort using balanced sampling to ensure equal representation of PDAC and MFP (55 cases each). This enriched design was adopted to ensure sufficient MFP cases for statistically meaningful comparison of radiologist performance with and without AI assistance. Each radiologist independently evaluated these cases across two reading sessions separated by a three‐week washout period to minimize recall bias. In the unassisted session, radiologists reviewed the CT images and clinical variables alone. In both reading sessions, radiologists were provided with the same set of clinical variables used as model inputs in addition to the CT images. In the AI‐assisted session, this interpretation was supplemented with the PancDS system's binary prediction and real‐time Grad‐CAM heatmaps highlighting discriminative regions. The order of cases was randomized for each radiologist in each session. All readers completed the unassisted session first, followed by the AI‐assisted session. During both sessions, readers were blinded to the histopathological results. For every case, a definitive diagnosis was recorded (Figure 7F).

### 4.10 Prospective Cohort and Evaluation Procedure

From January to October 2025, we conducted a prospective observational study at Tongji Hospital with consecutive enrollment (Cohort H). Adult patients (≥18 years) clinically suspected of PDAC who underwent contrast‐enhanced CT prior to any oncologic treatment were eligible. Only cases with histopathological confirmation enabling definitive classification as PDAC or MFP were included. Exclusion criteria followed those used for the retrospective cohorts (Figure 7 and Section S3), with two additional exclusions for the prospective cohort: (i) histopathology not confirming PDAC or MFP, and (ii) absence of contrast‐enhanced CT within 30 days prior to pathology. Prior to prospective validation, PancDS was completely frozen; no aspect of the model—including its architecture, training‐derived normalization statistics, selected feature weights, or the operational probability threshold—was updated, retrained, or calibrated using the prospective data. CT images and clinical variables were processed using the same pipeline as in the retrospective cohorts, and model outputs were compared against histopathology.

### 4.11 Statistics

All statistical analyses were performed using Python (version 3.8). Continuous variables were compared using the Student's *t*‐test for normally distributed data or the Mann‐Whitney *U* test for non‐normally distributed data. Categorical variables were analyzed using the Chi‐squared test or Fisher's exact test, as appropriate. Model performance was evaluated using receiver operating characteristic (ROC) curves and precision‐recall curves. The DeLong test was used for pairwise comparison of area under the curve (AUC). Bootstrap resampling with 1000 iterations was employed to estimate 95% confidence intervals for AUC values. Decision curve analysis (DCA) was employed to assess clinical utility. In the reader study, the improvement in diagnostic performance with AI assistance was assessed using McNemar's test for paired binary data. Bootstrap resampling with 1000 iterations was employed to calculate 95% confidence intervals (CI) for the improvements in accuracy, precision, sensitivity, and specificity. A *P* value <0.05 was considered statistically significant.

## Funding

This work is supported by the grants from National Key Research and Development Program of China No.2024YFC2419300 and National Natural Science Foundation of China (NSFC) No.82371942/82202127/62131009.

## Ethics Statement

This multicenter retrospective study was approved by the Institutional Review Board of Tongji Hospital, Tongji Medical College, Huazhong University of Science and Technology (Approval No.TJ‐IRB202506060)

## Consent

For the retrospective component of this study, the requirement for written informed consent was waived by the institutional ethics committees due to the retrospective use of de‐identified data. For the prospective non‐interventional component, written informed consent was obtained from all participants prior to enrollment.

## Conflicts of Interest

The authors declare no conflicts of interest.

## Supporting information




**Supporting File**: advs75747‐sup‐0001‐SuppMat.docx.

## Data Availability

The data that support the findings of this study are available from the corresponding author upon reasonable request.
