# Supplementary material for: PancDS in Real‐World Practice: A Prospective Multicenter Validation of a Clinical Decision‐Support System Bridging Experience Gaps in Pancreatic Lesion Diagnosis
Source: Adv Sci (Weinh). 2026 May 19;13(45):e75747. doi: 10.1002/advs.75747 (PMC13335980; doi:10.1002/advs.75747)
Supplement: Supplementary file 1 [file ADVS-13-e75747-s001.docx]

**Supporting Information**

**Contents**

[1. Calculating the importance of clinical features	2](#_Toc228031084)

[2. Subgroup analysis for PancDS	3](#_Toc228031085)

[3. Patient inclusion and exclusion criteria	4](#_Toc228031086)

[4. Semi-automatic segmentation	5](#_Toc228031087)

[5. Radiomics feature extraction and feature selection	6](#_Toc228031088)

[6. PANet Deep learning network and feature extraction	8](#_Toc228031089)

[7. TriFusionNet structure and weight calculation method	11](#_Toc228031090)

[Supplementary Table S1: Patient demographics of external test cohort and prospective cohort	12](#_Toc228031091)

[Supplementary Table S2. Comparison of different fusion strategies	13](#_Toc228031092)

[Supplementary Table S3. Detailed information on the major scanner models	14](#_Toc228031093)

**1. Calculating the importance of clinical features**

In this study, we employed a quantitative approach to evaluate the importance of clinical parameters relevant for differentiating PDAC from MFP using a fully connected neural network model. The significance of each parameter was assessed using the Integrated Gradients method from Captum, a library for model interpretability in PyTorch. The Integrated Gradients attribution for each clinical parameter $x_{i}$ is calculated as:

$$\mathrm{IG}_{i}\left( x \right)=\left( x_{i}-x_{i}^{'} \right)\times\int_{\alpha=0}^{1} \frac{\partial F(x^{'}+\alpha(x-x^{'}))}{\partial x_{i}}d\alpha$$

where $x^{'}$ denotes a baseline input (set to zero in our implementation), $x$ is the actual input, and $F$ is the model's output function. The integral captures the cumulative effect of varying parameter $i$ along the straight-line path from the baseline to the actual input.

The overall importance of parameter $i$ was then computed across all input samples:

$${Importance}_{i}=\frac{1}{N}\sum_{K=1}^{N} \left| \mathrm{IG}_{i}(x^{(k)}) \right|$$

where $N$ is the total number of samples. This approach allows for a comprehensive evaluation of each clinical parameter's contribution to the model's predictions, ensuring an enhanced understanding of the critical factors influencing the differentiation between PDAC and MFP, thereby facilitating informed clinical decisions and maintaining transparency in our model's predictive capabilities.

**Supplementary Figure S1.** (A) feature importance ranking plot; (B) spearman correlation heat map of the selected features.

**2. Subgroup analysis for PancDS**

To further assess the robustness of PancDS across different patient populations and lesion characteristics, we conducted subgroup analyses stratified by sex, age, and tumor size. Sex was dichotomized into male and female subgroups. For age, 60 years was chosen as the cutoff, as it approximates the median onset age of PDAC reported in epidemiological studies and is widely adopted as a clinically meaningful threshold for pancreatic malignancy risk stratification. For tumor size, a 3 cm diameter threshold was applied, consistent with the AJCC staging criterion distinguishing T1/T2 PDAC lesions, which also represents a clinically relevant boundary for diagnostic difficulty since smaller lesions are more prone to misclassification. Considering the relatively low incidence of MFP and that further stratification within a single cohort would substantially reduce the minority-class sample size and introduce considerable result variability, we merged the internal test cohort (Cohort B) and the external test cohort (Cohort G) into a combined cohort to enhance the statistical stability of the subgroup evaluation. As shown in Supplementary Figure S2, PancDS maintained consistently strong discriminative performance across all subgroups, with comparable AUCs between male (0.896, 95% CI: 0.851–0.936) and female (0.898, 95% CI: 0.792–0.966) patients, between younger (<60 years: 0.925, 95% CI: 0.878–0.963) and older (≥60 years: 0.858, 95% CI: 0.789–0.917) patients, and between smaller (<3 cm: 0.867, 95% CI: 0.797–0.930) and larger (≥3 cm: 0.939, 95% CI: 0.900–0.970) lesions, indicating that the diagnostic capability of PancDS is not substantially affected by sex, age, or tumor size.

**Supplementary Figure S2.** ROC curves of PancDS on the combined test cohort (Cohort B + Cohort G) stratified by (A) sex, (B) age, and (C) tumor size.

**3. Patient inclusion and exclusion criteria**

Inclusion criteria: (1) Confirmed by surgical pathology or puncture pathology as PDAC; (2) Confirmed by surgical pathology as MFP; (3) Perform CT enhanced scan within 30 days before surgery and have complete clinical data; (4) For PDAC, preoperative CT shows no contact between the pancreatic mass and the large artery, no contact with the superior mesenteric vein or portal vein, or no ≤ 180° contact, and no irregular venous contours; (5) For MFP, preoperative CT showed focal lesions of the pancreas with abnormal density and clear contours. The preoperative diagnosis was possible pancreatic ductal carcinoma, which cannot be ruled out.

Exclusion criteria: (1) inadequate CT quality or incomplete clinical documentation; (2) PDAC with distant metastases; (3) prior neoadjuvant therapy or corticosteroid treatment; (4) history of other malignancies within 5 years preceding PDAC/MFP diagnosis. Tongji Hospital, Tongji Medical College, Huazhong University of Science and Technology retained 634 eligible cases (538 PDAC, 96 MFP), randomly allocated in a 7:3 ratio to training (376 PDAC, 67 MFP) and internal test (162 PDAC, 29 MFP) cohorts. The external test cohort comprised 372 cases (297 PDAC, 75 MFP) from other 4 hospitals.

**4. Semi-automatic segmentation**

The pipeline commenced with training a nnUNetv2 model on venous-phase CT images from the Medical Segmentation Decathlon (MSD) dataset, utilizing 3D full-resolution configuration across 1,000 training epochs. Given the substantial imaging similarity between PDAC and MFP, the pre-trained model was directly applied to generate preliminary segmentation masks for all study cases.

To ensure anatomical accuracy, two board-certified abdominal radiologists (with 10 and 8 years' experience respectively) independently refined the automated segmentations. Segmentation agreement between the two radiologists was assessed using the Dice similarity coefficient. Masks with Dice > 0.90 were directly retained, whereas the remaining cases underwent consensus review and manual revision by the two radiologists to generate the final masks used for subsequent model training. This hybrid approach combining automated segmentation with expert verification achieved optimal balance between efficiency and precision.

**Supplementary Figure S3.** Semi-automatic segmentation flow chart

**5. Radiomics feature extraction and feature selection**

In this study, the pyradiomics library was utilized for radiomic feature extraction to analyze the characteristics of the pancreas or pancreatic tumors in venous-phase enhanced CT images. To ensure the accuracy and consistency of the feature extraction, several parameters were set: binWidth=25, Interpolator=sitk.sitkBSpline, resampledPixelSpacing = (1, 1, 1), and normalize=True. During the image preprocessing, feature extraction was performed on the original images, logarithmically transformed images (using sigma parameters of [1.5, 3.0, 5.0]), wavelet transformed images, and gradient images. This series of image transformations enabled capturing feature information at different scales and frequencies within the images. The specific radiomic features extracted included shape features, first-order statistics, Gray-Level Co-occurrence Matrix (GLCM) features, Gray-Level Run Length Matrix (GLRLM) features, Gray-Level Size Zone Matrix (GLSZM) features, Neighboring Gray-Tone Difference Matrix (NGTDM) features, and Gray-Level Dependence Matrix (GLDM) features, based on the tumor mask, resulting in a total of 1,236 features. After feature extraction, cohort-specific standardization was performed on all features encompassing both pancreatic cancer and mass-forming pancreatitis to mitigate the impact of extreme values on modeling. This step ensured a balanced contribution of each feature to the model. In the feature selection process, univariate t-tests were first applied to eliminate non-significant features. Subsequently, LASSO regression with 10-fold cross-validation was employed to further refine the feature set, selecting the six most robust features for the final signature. Through this systematic approach to feature extraction, standardization, and selection, critical information was effectively captured from the images, providing a reliable feature set for subsequent radiomic analyses and modeling. This process ensured the stability of the features and the accuracy of the models, laying a solid foundation for the diagnosis and prediction of pancreatic diseases.

The six selected radiomic features included：

original_shape_Maximum2DDiameterSlice；

original_glcm_JointAverage；

log-sigma-3-0-mm-3D_glrlm_RunLengthNonUniformity；

log-sigma-3-0-mm-3D_glszm_GrayLevelNonUniformity；

wavelet-LLL_glszm_GrayLevelNonUniformity；

gradient_glcm_Imc2

These features play significant roles in distinguishing PDAC from MFP. Each of these features captures different aspects of the tumor's characteristics that can assist in accurate diagnosis. For instance, original_shape_Maximum2DDiameterSlice provides insights into tumor size, which tends to be larger and more irregular in PDAC compared to the less pronounced dimensions of inflamed tissues in MFP.

The subsequent features enhance the analysis by focusing on texture and intensity relationships within the tumors. The original_glcm_JointAverage reflects the average intensity of voxel pairs, with higher values potentially indicating the denser cellularity typical of PDAC. Meanwhile, log-sigma-3-0-mm-3D-glrlm-RunLengthNonUniformity and log-sigma-3-0-mm-3D-glszm-GrayLevelNonUniformity measure the consistency of texture in PDAC, highlighting the differences from the more heterogeneous texture associated with MFP. Additionally, the wavelet-LLL_glszm_GrayLevelNonUniformity captures variability at multiple scales, providing nuanced insight into tissue architecture. Lastly, gradient_glcm_Imc2 can reveal structural changes in tumor vasculature that are more pronounced in PDAC. Together, these features facilitate a multidimensional approach to classifying pancreatic diseases, greatly improving diagnostic accuracy and aiding in the development of predictive models.

**6. PANet Deep learning network and feature extraction**

**6.1 Image preprocessing and input construction**

Solid pancreatic masses on portal-venous-phase CT were first segmented with a pretrained nnUNetv2 model, and the smallest 3D bounding box enclosing each predicted mask was cropped from the original volume, leaving a thin margin of surrounding tissue. Because lesion size varied widely across patients, every cropped volume was resampled to a fixed shape of 32 × 224 × 224 voxels (depth × height × width). Intensities were then z-score normalized using the mean and standard deviation computed inside the lesion mask, rather than over the whole volume, to limit the influence of surrounding fat and bowel gas. Cases in which the automatic segmentation produced no connected component, or a mask thinner than 8 voxels along any axis, were excluded from training and testing, with no imputation.

**6.2 Network architecture**

PANet takes a single-channel 32 × 224 × 224 volume as input. The stem is a 3 × 7 × 7 convolution with stride (1, 2, 2), which halves the in-plane resolution, keeps the slice dimension intact, and lifts the feature map to 16 channels.

The backbone consists of three sequential blocks. Each block contains a standard ConvBlock (Conv3d → BatchNorm3d → ReLU, 3 × 3 × 3 kernel) followed by a residual unit of two stacked ConvBlocks with an identity shortcut. Downsampling is controlled by the stride of the first ConvBlock: (1, 2, 2) in stage 1 to preserve slice-wise context, and 2 in stages 2 and 3. Channel width grows as 16 → 32 → 64 → 128, so early layers stay compact while deeper ones have the capacity to encode more abstract patterns.

Three attention modules are applied after the backbone in a fixed order: channel, spatial, and lesion-aware. The channel branch follows a squeeze-and-excitation design: global average and max pooling are fed in parallel into a shared two-layer MLP with a reduction ratio of 16, summed, and passed through a sigmoid to produce per-channel weights. The spatial branch concatenates channel-wise max- and average-pooled maps and processes them with a single 7 × 7 × 7 3D convolution to generate a spatial attention map. The lesion-aware branch, which we added specifically for this task, downsamples the binary segmentation mask to the current feature-map resolution with nearest-neighbor interpolation, converts it into a learnable embedding, and multiplies it element-wise with the feature map, forcing the network to keep attending to the lesion boundary and its internal heterogeneity even after repeated downsampling.

**6.3 Training configuration**

The model was trained with Adam (weight decay 1 × 10⁻³, batch size 16) for 400 epochs, using cosine annealing with T_max = 100 to decay the learning rate from 5 × 10⁻⁴ to 1 × 10⁻⁵. Because PDAC outnumbered MFP by roughly 4.7 : 1 in our cohort, we used Focal Loss in place of cross-entropy, with α = 0.75 and γ = 2.0 selected by a short grid search on the training set. Convolutional layers were initialized with Kaiming normal, fully-connected layers with a normal distribution (σ = 0.01), and BatchNorm layers with weight 1 and bias 0; dropout of 0.3 was placed before the classifier. Checkpoints were saved every 40 epochs, and the one with the highest AUC on the internal validation set was kept as the final PANet, with no separate early-stopping rule. All code was implemented in PyTorch 2.0 and trained on a single NVIDIA GPU with CUDA.

**6.4 Feature extraction**

Feature evolution through PANet follows the usual hierarchy of 3D CNNs: shallow layers respond mainly to edges and local texture, mid-level blocks pick up anatomical structures such as pancreatic duct contours and the lesion–parenchyma interface, and deeper blocks combine these cues into more abstract descriptors related to tumor–stroma interaction and intra-lesion heterogeneity. The residual shortcut in every block preserves fine-grained shallow information as the receptive field grows and keeps gradient flow stable during backpropagation. After the last attention block, adaptive 3D average pooling to 1 × 1 × 1 collapses the spatial dimensions into a fixed-length vector, which a fully-connected layer then projects to 64 dimensions for use as the deep feature representation in all downstream analyses—large enough to stay discriminative, small enough to remain stable when fused with radiomic and clinical features. The overall architecture is summarized in **Supplementary Figure S4**.

**Supplementary Figure S4.** PANet structure diagram. FCN, fully connected neural network.

**7. TriFusionNet structure and weight calculation method**

The fusion mechanism introduces a contribution-aware weighting protocol. Modality-specific diagnostic performance was quantified exclusively using the internal validation cohort (Cohort B), yielding the following AUC values: clinical model AUC = 0.76, radiomics model AUC = 0.87, and PANet model AUC = 0.91. These internal validation AUCs were used to compute fusion weights (wᵢ) and dimension allocations (dᵢ) as static hyperparameters. Importantly, no information from the external test cohorts (Cohorts C–G) or the prospective cohort (Cohort H) was used in weight determination, ensuring complete independence of external validation.

The weighted feature subspaces are subsequently projected into unified 256-D representations through adaptive fully-connected layers with dimension-specific dropout (clinical: 92-D, radiomic: 52-D, deep: 112-D). This quantified weighting scheme prioritizes highly discriminative features while preserving modality-specific information integrity. The final diagnosis from the PancDS system was determined by applying a pre-defined threshold to the model's output probability score. A score greater than 0.4 was classified as MFP, while a score of 0.4 or less was classified as PDAC.

𝑊e converted the AUC of each feature modality into an unnormalized, non-negative weight numerator $z_{i}$:

$$z_{i}=\left( \frac{AUC-0.5}{0.5} \right)^{2}$$

Then we normalized all weight numerators to obtain the final proportional weights *w_i_*:

$$w_{i}=\frac{z_{i}}{\sum_{j=1}^{n} z_{j}}=\frac{\left( \frac{{AUC}_{i}-0.5}{0.5} \right)^{2}}{\sum_{k=1}^{n} \left( \frac{{AUC}_{k}-0.5}{0.5} \right)^{2}}$$

We applied these proportional weights to allocate dimensions in the fusion model, assigning each feature modality a dimensionality of: $d_{i}=D\cdot w_{i}$ and $\sum_{i=1}^{n} d_{i}=D$，where D=256

**Supplementary Figure S5.** TriFusion structure diagram. FCN, fully connected neural network.

**Supplementary Table S1: Patient demographics of external test cohort and prospective cohort**

| Characteristics | External test cohort (Cohort G) | | | Prospective cohort (Cohort H) | | |
| --- | --- | --- | --- | --- | --- | --- |
|  | PDAC | MFP | *P* value | PDAC | MFP | *P* value |
| Age (years) | 63.52±10.52 | 57.78±10.64 | <0.001 | 61.95±10.02 | 59.20±13.41 | 0.332 |
| Sex |  |  | <0.001 |  |  | 0.279 |
| Male | 162 | 18 |  | 80 | 4 |  |
| Female | 132 | 60 |  | 56 | 11 |  |
| CA19-9 (U/mL) | 709.07±1345.58 | 59.72±144.99 | <0.001 | 703.32±1533.57 | 20.01±18.60 | 0.087 |
| Amylase (U/L) | 129.40±491.71 | 105.88±137.79 | 0.676 | 256.46±1050.86 | 80.90±43.03 | 0.520 |
| Lipase (IU/L) | 117.92±861.75 | 206.41±517.46 | 0.781 | 335.82±705.62 | 457.43±987.31 | 0.545 |

*Note:* *P* values were calculated by comparing each cohort with the training cohort.

**Supplementary Table S2. Comparison of different fusion strategies**

| Dataset | Metrics | AttentionFusion | WeightedAverage | SimpleConcate | TriFusionNet |
| --- | --- | --- | --- | --- | --- |
| Internal test cohort  cohort B | AUC (95% CI) | 0.940(0.873-0.989) | 0.932(0.863-0.990) | 0.928(0.844-0.992) | 0.936 (0.864-0.993) |
|  | Accuracy (%) | 90.1 | 91.1 | 92.1 | 92.7 |
|  | Recall (%) | 86.2 | 85.0 | 85.0 | 86.2 |
|  | Precision (%) | 62.5 | 65.8 | 69.4 | 71.4 |
|  | Specificity (%) | 90.7 | 92.0 | 93.1 | 93.8 |
| External test cohort  cohort G | AUC (95% CI) | 0.849(0.791-0.902) | 0.858(0.801-0.908) | 0.872(0.828-0.912) | 0.881 (0.833-0.924) |
|  | Accuracy (%) | 86.6 | 85.8 | 83.6 | 87.4 |
|  | Recall (%) | 65.3 | 66.7 | 56.0 | 68.0 |
|  | Precision (%) | 67.1 | 58.7 | 60.0 | 68.9 |
|  | Specificity (%) | 91.9 | 92.6 | 90.6 | 92.3 |

Note: Class labels were defined as PDAC = 0 and MFP = 1. ROC = receiver operating characteristic, AUC = Area Under the Curve, CI = Confidence Interval.

| CT scanners | GE | GE | Canon | Toshiba | Canon | Siemens | Philips | GE | GE |
| --- | --- | --- | --- | --- | --- | --- | --- | --- | --- |
|  | Discovery CT750 HD | Revolution CT | Aquilion One Vision | Aquilion 64 | Aquilion Prime | SOMATOM Drive | Brilliance iCT 256 | Revolution CT | LightSpeed VCT |
| Tube voltage, kVp | 120 | 120 | 120 | 120 | 120 | 120 | 120 | 120 | 120 |
| Tube current, mA | 200 | 150 | 200 | 160 | 200 | 200 | 150 | 150 | 200 |
| Pitch | 0.984 | 0.813 | 0.813 | 0.984 | 0.828–0.844 | 0.8 | 0.915 | 0.813 | 0.813 |
| Slice thickness, mm | 0.625 | 1 | 1 | 1 | 1 | 1 | 1 | 1 | 1 |
| Contrast agent | Ultravist 370 | Ultravist 370 | Ultravist 370 | Ultravist 370 | Ultravist 370 | Ultravist 370 | Iohexol 350 | Ultravist 370 | Ultravist 370 |
| Contrast dose (mL/Kg) | 1.0 | 1.5 | 1.5 | 1.5 | 1.5 | 1.2 | 1.5 | 1.5 | 1.5 |
| Contrast injection rate (mL/s) | 2.5-3.0 | 3 | 3 | 3.0 | 3 | 3 | 3 | 3 | 2.5-3.0 |
| Pancreatic phase (s) | 40-50 | 40-50 | 40-50 | 40-50 | 40-50 | 45 | 40-50 | 40-50 | 40-50 |
| Venous phase (s) | 60-70 | 65 | 65 | 60-80 | 65 | 70 | 65 | 65 | 65 |

**Supplementary Table S3. Detailed information on the major scanner models**
